# Oral Hygiene, Dietary Habits, and Saliva Properties in Relation to the Decayed, Missing, and Filled Teeth Index of Dental Students: A Pilot Study

**DOI:** 10.3390/medicina60122023

**Published:** 2024-12-08

**Authors:** Zoran Mandinić, Jelena Stojanović, Aleksandra Prokić, Miloš Beloica, Svetlana Jovanović, Jovana Kuzmanović Pfićer, Rasa Mladenovic, Dušan Kosanović, Milena Milanović, Miloš Todorović, Maja Milošević Marković, Ivan Dožić

**Affiliations:** 1 Clinic for Pediatric and Preventive Dentistry, School of Dental Medicine, University of Belgrade, 11000 Belgrade, Serbia; zoran.mandinic@stomf.bg.ac.rs (Z.M.); jstojanovic142@gmail.com (J.S.); aleksandra_prokic@yahoo.com (A.P.); milos.beloica@stomf.bg.ac.rs (M.B.); dusan.kosanovic@stomf.bg.ac.rs (D.K.); milena.milanovic@stomf.bg.ac.rs (M.M.); milos.todorovic@stomf.bg.ac.rs (M.T.); 2 Department of Public Health, School of Dental Medicine, University of Belgrade, 11000 Belgrade, Serbia; maja.milosevic@stomf.bg.ac.rs; 3 Department for Medical Statistics and Informatics, School of Dental Medicine, University of Belgrade, 11000 Belgrade, Serbia; jovana.kuzmanovic@stomf.bg.ac.rs; 4 Faculty of Medical Sciences, University of Kragujevac, 34000 Kragujevac, Serbia; 5 Department of Biochemistry, School of Dental Medicine, University of Belgrade, 11000 Belgrade, Serbia; ivan.dozic@stomf.bg.ac.rs

**Keywords:** DMF index, dental students, dental caries, oral health, diet, food and nutrition, preventive dentistry

## Abstract

*Background and Objectives*: Caries development is associated with poor oral hygiene, inadequate dietary habits, quantitative and qualitative food content, and a high level of bacterial plaque. Physical and chemical changes in saliva composition and particularly changes in its buffering capability play a significant role in caries development. This study aimed to determine the predictors of poor oral health among a sample of second-year dental students. *Materials and Methods*: The oral health of students was estimated with clinical examination and the DMFT index. The interview included questions about demographics status (gender, age), a dietary habits questionnaire, questions about oral hygiene, and a demonstration of tooth brushing technique. Unstimulated saliva samples were collected to measure buffering capacity and pH. *Results*: Almost half of the students have a low level of caries experience (46.7%), followed by a slightly smaller number having a medium level (41.3%), while one in eight students (12.0%) demonstrates a high level of caries experience. Male students have a statistically higher DMFT index 5.7 ± 1.2 than female participants 4.5 ± 0.5 (*p* = 0.036). Significant predictors for the DMFT index score in students were milk [β-coefficient = −0.338, *p* = 0.011] and yoghurt [β-coefficient = 0.284, *p* = 0.040] consumption. All students brushed their teeth two or more times during the day, usually after waking up before breakfast and before bedtime. Most students (85.3%) apply the proper tooth brushing technique, with female students applying it more frequently (*p* = 0.038). The mean salivary pH was 6.9, while the mean buffer capacity was 5.8. Female respondents have a statistically lower buffer capacity (5.8 ± 0.5) than male respondents (6.1 ± 0.4) (*p* = 0.047). *Conclusions*: Dentistry students are introduced to good oral hygiene habits, especially female students, while dietary habits should be improved. However, one in eight dental students is at high caries risk. By assessing the level of caries experience, targeted strategies can be developed to promote, maintain, and enhance oral health among dental students.

## 1. Introduction

Students are regarded as a vulnerable group when speaking of oral health-related knowledge, habits, and behavior patterns. Considering that physicians’ health practices strongly influence patient health practices, the oral health and dietary habits of dental students, future dentists, are extremely important and deserve special attention [1].

Dietary habits, the number of nutrients in food, oral hygiene habits, and the level of bacterial plaque are linked to oral health. Dietary habits are defined as the set of routine actions which lead people to select, consume, and use certain foods or diets in response to social and cultural influences [2]. The evolution of lifestyle has triggered changes in dietary habits among students and the public. Frequent snacking, sugary foods and drinks, and a diet rich in carbohydrates and lipids could lead to a rise in the carious process, especially if accompanied by inadequate oral hygiene [3].

Saliva plays a vital role in oral health [4]. It protects the teeth and oral mucosa by providing a favorable environment for the natural microflora, and facilitates speech, mastication, and swallowing [5]. The salivary buffering capacity is an important factor in dental caries resistance. A buffered system resists the changes in pH that occur with the formation of acidic and basic ions. When the buffering capacity is overwhelmed, and the balance of mineralization is undermined, pH levels decline, which promotes increased demineralization and the appearance of cavities [6].

Dental caries is defined as a biofilm-mediated, diet-modulated, multifactorial, non-communicable, dynamic disease resulting in net mineral loss of dental hard tissues. It is determined by biological, behavioral, psychosocial, and environmental factors. As a consequence of this process, a caries lesion develops [7,8,9]. The pathological process begins in the biofilm, where bacteria break down carbohydrates into organic acids, reducing the pH and leading to the demineralization of dental tissues [10,11]. The development of caries is related to the interaction of bad dietary habits, the quantitative composition of food consumed, inadequate oral hygiene habits, and prominent levels of bacterial deposits [12]. Physical and chemical changes in the content of saliva, especially changes in the buffering capacity of saliva, play a significant role in the development of caries [13].

Second-year students are at the beginning of college, they have just become independent from their parents, and thus they are forced to take care of their everyday nutrition. The data show that healthy behavior declines dramatically during the transition to young adulthood [14], and that inadequate dietary habits can also be encountered among students of medical disciplines [15,16]. Better understanding of the oral health and dietary habits of dental students can be of great help for dentists. That knowledge can help practitioners to provide better education to their patients about maintaining proper oral health.

In light of this background, the aim of the study was to identify the predictors of poor oral health among a sample of second-year dental students.

## 2. Materials and Methods

### 2.1. Population Understudy

A cross-sectional study was conducted at the School of Dental Medicine University of Belgrade, in October and November 2022. The Ethical Committee approved this study of the School of Dental Medicine, Belgrade University (No. 36/8).

The second-year students attending the course Preventive Dentistry in the first week of the winter semester were selected in order of appearance. The eligibility criteria for students included the following: being at least 18 years old, attendance in the Preventive Dentistry course, willingness to participate in the study, and a signed informed consent form. The exclusion criteria included the following: under 18 years of age, chronic diseases, and smokers.

Before the beginning, the study’s objectives and the entire procedure were thoroughly presented to the participants, after which written consent was obtained.

### 2.2. The Procedure of Dental Examination

The data in this study were obtained by dental examination according to the World Health Organization (WHO) criteria [17]. A dental examination was performed by a dental faculty member previously calibrated (Z.M.) and one fifth-year dental student (Inter-examiner agreement was achieved (Kappa: 0.72)). The dental office at the Clinic for Pediatric and Preventive Dentistry, School of Dental Medicine, University of Belgrade, facilitated all dental examinations. The dental office was equipped with a dental chair, adequate light, and standard dental instruments. All partly or fully erupted permanent teeth were examined except for the third molars. The decayed/missing/filled teeth index (DMFT) was used to evaluate the dental caries prevalence among students. It is used to keep track of the decayed (D), missing (M), and filled (F) teeth. The DMFT values are interpreted according to the DMF scoring scale. According to this scale, a DMFT value between 0 and 4 is considered low, a value in the range of 5–9 is moderate, and a value greater than 9 is a high level of caries experience [18].

### 2.3. Survey

After the dental examination, each participant was assessed using a structured interview. The interview included questions about demographics status, a dietary habits questionnaire [19,20], questions about oral hygiene, and a demonstration of a proper tooth brushing technique. The demographic questionnaire contained questions about gender and age. A dietary habits questionnaire was part of the overall questionnaire, previously used in the national survey “Serbian Population Health Survey 2019” by the Republic Institute of Public Health of Serbia ‘Dr Milan Jovanović Batut, which was conducted in accordance with the methodology of the European Health Interview Survey (EHIS, Wave 3), adapted to the Serbian population [19,20]. The dietary habits questionnaire is a brief one-dimensional instrument and contains 12 questions related to the consumption of the following food products: coffee, carbonated beverages, non-carbonated beverages, additionally sweetened beverages such as coffee or tea, sweets, meals containing meat, meat-free food, fruit, vegetable, milk, cheese, and yogurt. Participants rate how often they consumed certain foods with answers offered on a 4-point Likert scale (never, a few times a week, once per day, a few times per day). The Cronbach’s alpha was 0.76, indicating a high level of internal consistency for this sample. Participants were asked about their oral hygiene practices, including the frequency of tooth brushing (less than twice a day/two or more times per day) and when they brush their teeth (after waking up before breakfast/after breakfast/after meals during the day/after dinner/before bedtime). Each participant demonstrated his or her tooth brushing technique in front of the dentist teacher who leads the Preventive Dentistry course, who then made a final determination of whether the technique was correct or not. The interview lasted around 30 min.

### 2.4. Saliva Samples and Sampling

The saliva’s pH value and buffer capacity were obtained by analyzing the collected saliva samples. The unstimulated saliva samples were collected at least 2 h after meals and at least 30 min after brushing teeth (between 10 am and 12 noon or 2 and 4 pm). Saliva samples were collected as part of a dental examination. Saliva was sampled using special tubes (Salivette^®^, Sarstedt, Nuembrecht, Germany) by placing a cotton pad on the floor of the mouth for 3 to 5 min. All samples were centrifuged at 3000 rpm for 10 min. The pH value of the saliva was measured at once after sampling using a pH meter (Martini Instruments, Riverside, USA). According to the modified Ericsson’s method, the buffer capacity of saliva was determined by adding 0.5 mL of HCl (0.005 mol/L) in 0.5 mL of each saliva sample. The salivary buffering capacity value was measured using a pH meter and ranked into one of the following three categories: low buffering capacity (pH < 4.5), medium buffering capacity (pH 4.5–5.5), and high buffering capacity (pH > 5.5) [21].

### 2.5. Statistical Analysis

All statistical analyses were performed using the Statistical Package for Social Science (SPSS software package, version 22.0; SPSS Inc., Chicago, IL, USA). The mean and standard error were used to describe the numerical data, while categorical data were described as frequencies and percentages. A one-sample Kolmogorov–Smirnov test was conducted to determine if their distribution was normal in numerical data. Parametric data were assessed using a T-test (Ph, buffer capacity, DMFT index according to gender) and one-way ANOVA (Ph, buffer capacity according to DMFT index). The Chi-square test was used to compare dietary habits according to gender, the DMFT index, and the brushing technique according to gender. Spearman’s Correlation Coefficient was used to show a correlation between dietary habits and the DMFT index.

The multivariate linear regression model was performed for the DMFT index as a dependent variable. Kappa statistics was used to measure author agreement [22]. Based on the study results, post hoc analysis was conducted in the G*power program (version 3.1.9.2.), and the achieved power was 99.38%. The power for 75 respondents was calculated for the difference between two independent means, α = 0.05, and the effect size was calculated according to the difference between two independent means and the SD (d = 1.03). The *p*-value was set as significant at the 0.05 level.

## 3. Results

Of the 90 students who met the criteria, 75 (83%) students participated, while 5 (6%) subjects filled out the questionnaires inadequately and 10 (11%) dropped out. Out of 75 participants, 60 (80%) were female, and 15 (20%) were male. The participants’ average age was 20 ± 0.8 years, aged from 19 to 22 years.

The results showed that 35 (46.7%) students had a low level of caries experience, 31 (41.3%) of them had a moderate level, while 9 (12.0%) had a high level of caries experience. The mean DMFT index of students was 4.7 ± 3.6. The male participants had a statistically higher DMFT index of 5.7 ± 1.2 than female participants, with 4.5 ± 0.5, respectively (*p* = 0.036).

The data on nutrition concerning the level of caries experience are shown in Table 1. Although the largest percentage of students who consume coffee daily, at least once, or more times per day belongs to the group of students with a high level of caries experience, there were no statistically significant differences between the groups. Students with a high level of caries experience consume carbonated beverages several times during the day more often than students with medium and low caries experience, but without statistical significance. The majority of participants reported consuming non-carbonated beverages rarely or a few times per week, with no statistically significant difference observed in relation to their level of caries experience. Even though the highest percentage of students who add sugar to their coffee and tea daily is found in the group of students with a high level of caries experience (57.7%), compared to students with medium (38.7%) and low levels (31.4), the difference was not statistically significant. A high frequency of daily consumption of sweets is present in all three groups of respondents, students with a low level of caries experience (91.4%), medium (100%), and least frequently by students with a high level of caries experience (88.9%), without statistical significance. Meat is highly prevalent in the daily diet of students, while meals without meat are consumed very rarely, with no significant differences between the groups. Daily fruit consumption (once or more times per day) was observed in more than half of the students with low and medium caries experience (57.2%, 51.6%, respectively), while it was slightly higher in the group of students with high caries experience (77.8%), but without statistical significance. The majority of students report consuming vegetables multiple times a day, with no statistically significant differences observed in relation to their level of caries experience. The frequency of consumption of dairy products (milk, cheese, and yogurt) was similar between all three groups, with no statistical differences.

Regarding maintaining oral hygiene, all students brushed their teeth two or more times during the day, usually after waking up before breakfast and before bedtime. Most of the students, 85.3%, apply the proper technique for tooth brushing. According to the values of the DMFT index, students with high values of the level of caries experience brush their teeth more irregularly (11.9%). Male dental students (30.8%) significantly more often have inadequate tooth brushing technique than female students (9.1%) (*p* = 0.038).

Salivary analysis presented those mean values of pH as 6.9 ± 0.2, while the mean buffer capacity was 5.8 ± 0.5. There is no statistically significant difference in the buffer capacity (*p* = 0.135) and pH value (*p* = 0.756) of saliva between groups with a low (pH = 6.9, buffer capacity = 5.8), medium (pH = 6.9, buffer capacity = 5.9), and high (pH = 6.8, buffer capacity = 5.5) level of caries experience. Furthermore, the results showed a statistically significant difference in buffer capacity among genders (*p* = 0.047), with female respondents having a lower value of 5.8 ± 0.5 than male respondents, with 6.1 ± 0.4.

The study found a statistically significant correlation between the consumption of milk and DMFT index score (rho = −0.258, *p* = 0.035). Other food did not show an important relationship with the DMFT index score (Table 2).

A linear regression analysis was conducted to identify dietary habits as potential predictors. Significant predictors for the DMFT index score in students were milk and yoghurt consumption (Table 3).

## 4. Discussion

Health professionals have an essential role in providing knowledge about oral health and its importance to public health. Dental students should aim to maintain a prominent level of awareness of self-oral health to influence patients and the community by example [23,24].

The present study showed that the mean DMFT index value in the dental students’ population is 4.7 ± 3.6, which is considered a low level of caries experience. Medical and dental students aged 18–25 years old from Bosnia and Hercegovina, Foca municipality, had an average DMFT value of 12.8 ± 4.7, which is significantly higher than the participants in our study [25]. Others have found that the DMFT index values were 4.5 for Spanish dental students [26] and 4.0 for Turkey dental students [27], which closely resembles the results obtained in our study. Another study reported a significantly lower value DMFT index among Israeli young adults (1.9) [28], while some others found a slightly higher value among dental students (6.0) [29].

Although Serbia might have a higher DMFT index value than some countries, the value has been continuously declining in recent years [30]. An increasing number of 25-year-olds in Serbia that have all teeth present (5.6% in 2006, 8.3% in 2013, 16.5% in 2019) can be influenced by improvements in oral hygiene [19]. Oral hygiene habits are continuously improving in the Serbian population, as according to the latest data from the national survey, 57.8% of people in Serbia brush their teeth more than once a day, with the highest percentage of regular tooth brushing found among young people aged 15–24 years [19]. The prevalence of tooth brushing at least twice a day among preclinical dental students in Lithuania is very high (92%) [31], which is in accordance with our results. And other studies have also showed a high percentage of dental students who brush their teeth at least twice a day [27,32]. In contrast, others have reported a somewhat lower prevalence of this desirable habit for maintaining adequate oral hygiene among dental students from the following Asian countries: Yemen (86.2%), India (78.3%), Pakistan (70%), and Saudi Arabia (65.2%) [15,23,29]. Some studies reported that female students brush their teeth more frequently twice a day than male students [23,24]. In contrast, others observed no difference in the frequency of tooth brushing based on gender among dental students [26,27]. Our study found no gender differences in the frequency of tooth brushing, but it showed that female dental students are more likely to use proper tooth brushing techniques compared to males.

Diet and nutrition also play a crucial role in caries susceptibility, both in the metabolism of dietary constituents by oral microorganisms and the modification of the host oral flora. Flavored drinks, especially sweetened carbonated beverages, have higher cariogenic potential due to their high sugar content and regular consumption [33]. Considering nutritional status as an important indicator of health, a previous study conducted on this student population revealed that one in nine dental students is overweight, while one in fourteen is underweight [34]. Studies have shown that the frequency of carbonated drink consumption is higher among individuals with high caries prevalence [35]. Although our results did not show a statistically significant difference in the consumption of sugary beverages in relation to the level of caries experience, the number of students who consume this carbonated beverage several times during the day was higher in the group of students with a high level of caries experience. The consumption of sweets in Serbia is very widespread. The projection for the consumption of sweetened products in Serbia, in 2020, in monthly quantities on average, per household member, is 1.001 kg [36]. Although a Serbian national study reports that the largest percentage of the population consumes sweets 1–2 times a week [19], which has also been reported in other studies [12,15], our results indicate a higher frequency of daily candy consumption among dental students. Students do not have access to a restaurant where they could eat properly, only the cafeteria, where sweets and sugary drinks are more accessible. However, no statistically significant higher level of caries experience was observed in these students, which can be explained by good habits in maintaining oral hygiene.

In the national diet profile among youth in Serbia, the daily consumption of fruits and vegetables has been reported to be similar to our findings, with a slightly higher frequency of vegetable consumption observed among the dental students in our sample [19]. This difference may be attributed to a higher level of health literacy regarding proper nutrition among students of medical professions compared to the general population. Although no statistical significance was observed, students with a high level of caries experience exhibited the highest frequency of fruit consumption in the presented study. A similar finding is reported in a study that indicates more frequent fruit consumption among young adults with high caries activity [37].

Serbia’s milk and dairy products consumption rate is 70 kg per capita annually, and cow milk is the most utilized and 42.6% of adults drink at least one cup of milk or milk products [38]. Students consume milk the rarest of all when it comes to dairy products, which is the opposite of some other study in which milk is the most often consumed of all dairy products [39]. It is well known that milk plays a positive role in hard tissue, where the bioactive compounds in milk contribute to reducing enamel demineralization and enhancing the remineralization process [40]. The lactose found in milk is not fermented like the other sugars, so it is less cariogenic because the phosphor proteins inhibit enamel dissolution. The milk’s antibacterial factors may interfere with the oral microbial flora [41]. In our study, it was shown that increased milk intake is associated with a high level of caries experience. There are studies that suggest milk has some cariogenic potential, making the oral cavity favorable for the development of acidogenic microorganisms [42]. One possible explanation could be that a cultural pattern in the Serbian population is that milk is most commonly consumed in combination with another food item, typically a sweet one. It has been noted that students do not have access to a proper restaurant where they can have a full meal, but only to a cafeteria where milk is often used as an accompaniment to coffee and tea.

Some of the possible explanations for the positive impact of yogurt on the lower level of caries experience is that yogurt contains probiotic bacteria, namely Lactobacillus bulgaricus and Streptococcus thermophilus, which act as inhibitors of cariogenic bacteria growth, and that the calcium content in yogurt helps in the tooth remineralization process [43]. Others also report a positive anticariogenic effect of yogurt in the studied populations [44,45]. Another possible explanation for the potential mechanisms underlying our observed inverse association between yogurt consumption and dental caries prevalence is the active action of casein phosphopeptides from yogurt. Several studies have demonstrated the anticariogenic properties of casein phosphopeptides, which stabilize high concentrations of amorphous calcium phosphate on the tooth surface, thereby preventing demineralization and promoting the remineralization of enamel caries [46,47,48]. Numerous studies have shown that bovine milk has a low cariogenic potential to provoke caries lesions in enamel [49,50].

Frequent consumption of sugar causes a decrease in the pH of the bacterial plaque, enabling the interaction of the substrate and microorganisms to reduce extracellular glucans. A continuous increase in acidogenic bacteria causes an imbalance in mineralization and the demineralization of dental tissues [51]. In this study, there was no difference between the pH value and buffer capacity in relation to the level of caries experience. Our results showed a lower buffer capacity among female students, which is in line with the results obtained regarding the frequency of tooth brushing. This assumption is confirmed by another study as well [52].

### Strengths and Limitations

A strength of this study is the observation that the majority of dental students, as future dental professionals, exhibit good oral health and maintain proper oral hygiene habits. On the other hand, students with poorer oral health tend to demonstrate inadequate tooth brushing techniques. Additionally, another strength of this study is the finding that foods such as milk are associated with a high level of caries experience, while yogurt is associated with a low level of caries experience.

A limitation of the study is the small sample size used for this pilot study, which only included second-year dental students, rather than all freshman years. Additionally, we did not consider other oral hygiene practices, such as fluoride use. Furthermore, we did not account for broader social determinants or other detrimental habits that may affect oral health (e.g., smoking, alcohol consumption, and the use of psychoactive substances), which are important for dental students to address in order to more effectively promote oral health in the future. We plan to expand the research to include a larger sample of students from all years of study, incorporating other risk factors that may influence oral health.

## 5. Conclusions

Based on the study’s findings, the majority of students have a low or medium level of caries experience, maintain good oral hygiene habits, and have slightly poorer dietary habits. However, one in eight dental students is at high caries risk. Female students exhibit better oral health status, more proper tooth brushing technique, and a lower buffering capacity. Milk has been proven to be a predictor of a high level of caries experience, while yogurt contributes to a low level of caries experience. By assessing the level of caries experience, targeted strategies can be developed to promote, maintain, and enhance oral health among dental students.

## Figures and Tables

**Table 1:** Dietary habits frequency in relation to the level of caries experience (*n* = 75).

Variables n (%)	Rarely or Never			A Few Times a Week			Once per Day			A Few Times per Day			*p*
**Level of caries experience**	Low	Medium	High	Low	Medium	High	Low	Medium	High	Low	Medium	High	
**Coffee consumption**	20 (62.5)	15 (48.4)	7 (75.0)	7 (20.0)	7 (22.6)	1 (11.1)	6 (17.1)	7 (22.6)	0 (0.0)	2 (5.7)	2 (6.4)	1 (11.1)	0.123
**Carbonated beverages consumption**	13 (45.5)	17 (54.8)	3 (33.3)	13 (45.5)	8 (25.8)	3 (33.3)	6 (17.1)	5 (16.2)	2 (22.2)	3 (8.6)	1 (3.2)	1 (11.1)	0.637
**Non-carbonated beverages consumption**	16 (45.8)	15 (48.4)	3 (37.5)	8 (22.8)	4 (12.9)	1 (11.1)	6 (17.1)	4 (12.9)	2 (22.2)	8 (22.8)	8 (25.8)	3 (37.5)	0.788
**Sweets consumption**	5 (14.3)	0 (0.0)	1 (11.1)	0 (0.0)	0 (0.0)	0 (0.0)	4 (11.4)	2 (6.4)	0 (0.0)	28 (80.0)	29 (93.6)	8 (88.9)	0.324
**Meals containing meat**	2 (5.7)	0 (0.0)	0 (0.0)	2 (5.7)	3 (9.7)	1 (11.1)	5 (14.3)	8 (25.8)	1 (11.1)	26(76.3)	20 (64.5)	7 (77.8)	0.670
**Meals without meat**	32 (91.4)	30 (96.8)	9 (10.0)	3 (8.6)	1 (3.2)	0 (0.0)	0 (0.0)	0 (0.0)	0 (0.0)	0 (0.0)	0 (0.0)	0 (0.0)	0.670
**Fruits consumption**	9 (25.7)	11 (35.5)	1 (11.1)	6 (17.1)	4 (12.9)	1 (11.1)	8 (22.9)	5 (16.1)	1 (11.1)	12 (34.3)	11 (35.5)	6 (66.7)	0.911
**Vegetables consumption**	1 (2.9)	1 (3.2)	0 (0.0)	3 (8.6)	4 (12.9)	1 (11.1)	8 (22.9)	10 (32.3)	2 (22.2)	23 (65.6)	16 (51.6)	6 (77.7)	0.926
**Milk consumption**	18 (51.4)	21 (67.7)	7 (77.8)	10 (28.5)	9 (29.1)	2 (22.2)	3 (8.6)	1 (3.2)	0 (0.0)	4 (12.5)	0 (0.0)	0 (0.0)	0.505
**Cheese consumption**	20 (57.2)	18 (58.0)	6 (66.7)	5 (14.3)	6 (19.4)	2 (22.2)	6 (17.1)	4 (12.9)	1 (11.1)	4 (11.4)	3 (9.7)	0 (0.0)	0.960
**Yogurt consumption**	11 (31.4)	13 (41.9)	2 (22.2)	13 (37.1)	11 (35.4)	3 (33.4)	9 (25.7)	6 (19.5)	1 (11.1)	2 (5.7)	1 (3.2)	3 (33.4)	0.364

Values are given as a number (percentage). *p* < 0.05 indicates statistical significance.

**Table 2:** DMFT index and dietary habits frequency.

	Spearman’s Rho Correlation Coefficient	Significance
Coffee consumption	−0.153	0.216
Carbonated beverages consumption	0.045	0.720
Non-carbonated beverages consumption	0.032	0.759
Additionally sweetened beverages such as coffee or tea consumption	−0.028	0.822
Sweets consumption	0.198	0.108
Meals containing meal	−0.155	0.211
Meals without meat	−0.034	0.786
Fruit consumption	−0.024	0.848
Vegetable consumption	−0.122	0.327
Milk consumption	−0.258	0.035 *
Cheese consumption	0.028	0.821
Yogurt consumption	0.080	0.519

* Significance: *p* ≤ 0.05.

**Table 3:** Results of linear regression to identify dietary habit predictors of DMFT index.

Model	B	Std. Error	Standardized Coefficient Beta	*p*	95% Confidence Interval Beta	
Carbonated beverages consumption	0.517	0.526	0.131	0.330	−0.537	1.571
Additionally sweetened beverages such as tea or coffee	0.098	0.369	0.034	0.792	−0.642	0.837
Sweets consumption	0.771	0.734	0.136	0.298	−0.700	2.242
Meals containing meat	−1.216	0.761	−0.227	0.116	−2.740	0.308
Meals without meat	−1.310	0.900	−0.220	0.151	−3.113	0.492
Fruit consumption	0.093	0.424	0.031	0.826	−0.755	0.942
Vegetable consumption	−0.487	0.571	−0.105	0.397	−1.630	0.656
Milk consumption	−1.598	0.607	−0.338	0.011 *	−2.813	−0.383
Cheese consumption	0.416	0.510	0.114	0.419	−0.606	1.437
Yogurt consumption	1.147	0.546	0.284	0.040 *	0.053	2.242

* Significance: *p* ≤ 0.05.

## Data Availability

The data presented in this study are available on request from the corresponding author.
